# The Differential Diagnosis of Multiple Glandular Swellings—II

**Published:** 1908-12-26

**Authors:** 


### Medicine.

### THE DIFFERENTIAL DIAGNOSIS OF MULTIPLE GLANDULAR SWELLINGS—II.

Another version is to consider Hodgkin's disease and lymphadenoma as synonymous terms, and to regard lymphosarcoma as something entirely different. The grounds for so doing seem insufficient, and meanwhile it is enough for clinical purposes to regard lymphadenoma, Hodgkin's disease, and lymphosarcoma as essentially the same disease, differing only in type as regards the spleen and as regards the acuteness of the fatal course.

It should be said in passing, however, that although there can be little doubt that there really is such a thing as Hodgkin's disease, it is diagnosed often enough when it is not present. Often glandular enlargement is diagnosed as Hodgkin's disease when the condition is really either lymphatic leuchæmia or tubercle—especially tubercle.

The characters presented by tuberculous glands, as a rule and in typical cases, are different from those presented by typical Hodgkin's disease glands, it is true. Not only are the tuberculous glands usually confined to the neck, thorax, or abdomen, but they are also, as a rule, matted together, adherent to one another and to the skin over them, the latter at one time or another becoming red and inflamed. The skin inflammation is due to secondary infection, softening and suppuration of the caseous tuberculous glands, and the result is the presence in the neck of depressed scars of former abscesses or one or more sinuses leading down to the site of a suppurating tuberculous gland. In typical cases of Hodgkin's disease, on the other hand, the enlarged glands are firm, non-adherent to one another or to the overlying skin, and they do not tend to soften or suppurate, so that reddening of the skin over them is very rare, and abscess formation or the presence of a sinus almost unknown. Whereas inflamed tuberculous glands are both tender and painful, in Hodgkin's disease glands are very seldom either. The masses of glands in Hodgkin's disease may become very large indeed, partly by increase in size of the individual glands, partly by the addition of new glands; tuberculous glands also enlarge progressively, but their mass seldom equals in size that of a well-marked case of Hodgkin's disease.

Nevertheless, it is most important to remember that in some cases in which the glandular enlargement is undoubtedly tuberculous, the characters of the enlarged glands are those described as typical of Hodgkin's disease; and yet the tuberculous nature of the lesion has been verified by microscopical examination of the glands. Some authorities have even gone so far as to say that all cases of so-called Hodgkin's disease are really tuberculous, but there is a serious objection to this view—namely, that, although some cases exhibit undoubted evidence of tubercle in the sections of the glands, others exhibit neither tubercle bacilli nor giant-celled systems, in spite of minute and careful examination. The future will show; but meanwhile it is generally conceded that there is a disease which is entitled to the name Hodgkin's disease as distinct from tubercle. The point is that the mere physical features of the glandular enlargement will not alone suffice for diagnosis.

*The Age of the Patient.*—If the glands are complained of for the first time in a person over forty years of age, and their enlargement is principally in the neck, secondary carcinomatous glands are always a possibility, and signs of epithelioma of the tongue, pharynx, or œsophagus should be looked for. Secondary malignant glands can sometimes be diagnosed even when no primary growth is found, partly by their rate of growth, but chiefly by their extreme hardness. In a patient under puberty a chronic enlargement of the glands in the neck is far more likely to be tuberculous than due to anything else. Lymphatic leuchæmia may occur at any age, but it is at least not less common in the young than it is in the middle-aged or elderly. Hodgkin's disease and its allies, on the other hand, are commoner in adults than they are in children.

*The Condition of the Spleen.*—An enlarged spleen in association with the enlarged glands favours a diagnosis of either Hodgkin's disease or lymphatic leuchæmia. The blood count will at once decide between these two. The arbitrary distinction drawn by some between lymphadenoma on the one hand and Hodgkin's disease upon the other is that Hodgkin's disease without an enlarged spleen is lymphadenoma. Enlarged cervical glands were observed to be present more than five years before there was any splenic enlargement in a case that was ultimately proved by post-mortem examination to be typical Hodgkin's disease. During the five years before the spleen became palpable the case would have been lymphadenoma and not Hodgkin's disease if the arbitrary rule were followed. The spleen does not enlarge in lymphosarcoma or in tuberculous glands, unless there is general tuberculosis or lardaceous disease.

*The Condition of the Mediastinal Glands.*—A careful examination of the thoracic physical signs may reveal distinct evidence of partial obstruction of a bronchus, presumably by enlarged bronchial or mediastinal glands of the same nature as those palpable in the neck, axillæ, or groins. When this is so, it becomes a fact of considerable importance to know that tuberculous glands, even those that have become quite big and caseous, practically never obstruct a bronchus. This has been confirmed again and again post-mortem. A tuberculous gland that might be big enough to obstruct a bronchus if it remained hard and resistant nearly always softens in a way that precludes obstructing power. Hence, if there is proof of considerable enlargement of intra-thoracic glands, it is probable that the case is not one of tubercle, but rather of Hodgkin's disease, lymphadenoma, lymphosarcoma, or secondary carcinoma. Lymphatic leuchæmia glands, though they do not suppurate, are usually soft; at any rate, they seldom obstruct a bronchus.

*Calmette's and von Pirquet's Tuberculo-reactions* are each of undoubted value in deciding whether a given case of glandular enlargement is tuberculous or not. Either Calmette's reaction, with 1 per cent. or 0.5 per cent. solution of Koch's old tuberculin upon the conjunctiva, or von Pirquet's reaction, with 25 per cent. tuberculin upon the abraded skin, if positive, indicates that the patient's body contains active tubercle bacilli.

*Excision of a Gland.*—This is no very serious matter if quite a superficial one be chosen. At the same time excision would not be resorted to in every instance. The main point that can be decided by it is whether the glands are tuberculous or not.

